# Characterisation of Morphic Sensors for Body Volume and Shape Applications

**DOI:** 10.3390/s20010090

**Published:** 2019-12-22

**Authors:** Sami El Arja, Titus Jayarathna, Ganesh Naik, Paul Breen, Gaetano Gargiulo

**Affiliations:** 1 School of Computing, Engineering and Mathematics, Western Sydney University, Penrith, NSW 2751, Australia; g.gargiulo@westernsydney.edu.au; 2 MARCS Institute for Brain, Behaviour and Development, Western Sydney University, Milperra, NSW 2560, Australia; Ganesh.Naik@westernsydney.edu.au (G.N.); P.Breen@westernsydney.edu.au (P.B.); 3 Translational Health Research Institute, Western Sydney University, Campbelltown, NSW 2560, Australia; 4 School of Psychiatry and Ingham Institute of Applied Medical Research, University of New South Wales, Liverpool, NSW 2052, Australia

**Keywords:** electro-resistive bands, flexible sensor, conductive fabric

## Abstract

Stretchable conductive materials are originally conceived as radio frequency (RF) and electromagnetic interference (EMI) shielding materials, and, under stretch, they generally function as distributed strain-gauges. These commercially available conductive elastomers have found their space in low power health monitoring systems, for example, to monitor respiratory and cardiac functions. Conductive elastomers do not behave linearly due to material constraints; hence, when used as a sensor, a full characterisation to identify ideal operating ranges are required. In this paper, we studied how the continuous stretch cycles affected the material electrical and physical properties in different embodiment impressed by bodily volume change. We simulated the stretch associated with breathing using a bespoke stress rig to ensure reproducibility of results. The stretch rig is capable of providing constant sinusoidal waves in the physiological ranges of extension and frequency. The material performances is evaluated assessing the total harmonic distortion (THD), signal-to-noise ratio (SNR), correlation coefficient, peak to peak (P-P) amplitude, accuracy, repeatability, hysteresis, delay, and washability. The results showed that, among the three controlled variables, stretch length, stretch frequency and fabric width, the most significant factor to the signal quality is the stretch length. The ideal working region is within 2% of the original length. The material cut in strips of >3 mm show more reliable to handle a variety of stretch parameter without losing its internal characteristics and electrical properties.

## 1. Introduction

Physiological monitoring and noninvasive sensing are paramount for the modern biomedical sensors industry. Recent technologies enable monitoring of vital physiological signals such as respiration, heart rate, electrocardiogram (ECG), and pulse velocity non-invasively. Non-invasive sensors enable comfortable yet accurate diagnosis and monitoring of sleep disordered breathing [1], peripheral vascular disease [2], and congestive heart failure [3], coronary artery disease [4], cardiopulmonary resuscitation [5] and cardiac arrhythmia [6]. Conventional portable monitoring sensors take advantage of recent advances of wireless networks technologies, mobile devices and cloud services to achieve a pervasive response [7]. Even with the current state-of-the-art technologies, end sensors remain moderately larger in size, cumbersome to use and unsuitable for long-term monitoring. Sleep studies are a good example scenario where long-term vital parameter monitoring is paramount. The sleep monitoring system should provide minimum intrusion to sleep [8,9]; however, the t current state-of-the-art Polysomnography (PSG) [10] is time-consuming, expensive, limited to in-clinic and provides minimum comfort. The alternative at-home portable sleep study devices use the same conventional sensors in smaller package. A radical change in sensor technologies required to make vital monitoring devices comfortable and “invisible" to the user. The rise of “invisibles” targets making wearable devices built into the commodities people use daily, such as clothes, shoes and jewellery [11]. Usage of the correct type of sensors would enable low-cost implementations that can be embedded in day-to-day clothes that facilitate long-term monitoring [12].

Recently, many sensors suitable for tidal monitoring have been presented [13,14,15,16,17]. Electro-resistive sensors based on carbon polymers have shown a great potential for biomedical sensors, and specifically wearable sensors [18], and bio-impedance monitoring [19]. Electro-resistive sensors are made by re-purposing electromagnetic interference (EMI) shielding fabric/gaskets which are mainly used for electrostatic discharge, shielding, power transmission and protect electronics from electromagnetic interference [20]. Using these special-purpose materials in wearable devices for health monitoring systems takes advantage of their flexibility and durability. Similarly, they have been used in robotics application for measuring displacement and force [21,22,23,24,25,26]. A thin cut from these materials produces an electro-resistive band (ERB), and ERB basically functions as distributed strain gauge with a gauge factor varying according to the speed of stretching, size and is also function of any additional protective coating. A careful understanding of this type of sensors is important to choose a band that can be suitable for detecting physiological parameters accurately for an extensive period of recording time.

Previous literature provides details regarding the performance and applications of different stretchable ERBs sensors. An attempt by Amjadi et al. [27] using a strain sensor made from nanocomposite materials made of silver nanowire and Polydimethylsiloxane (PDMS) elastomer was characterised based on their stretchability, hysteresis performance and bendability. Their method shows high linearity and negligible hysteresis. Lorussi et al. [18] reported using elastic fabric covered with an epitaxial layer of conducting polymer which was characterised in terms of quasi-static and dynamic electro-mechanical transduction properties, and thermal and aging properties. The results showed that these fabrics can be used to integrate them into wearable instrumented garments. Fernández-García et al. [28] investigate the characterisation of fabric with conductive and non-conductive zones and, based on their strategies, the fabric is more comfortable in wearable technologies. Charn and Mamun [29] investigated the physical and electrical characteristics of different textile materials to be used for capacitance biosensors used in clothing, bedding, and wearable items. These studies have tested with a wide range of the electro-resistive and electro-capacitive bands, however, the characterisations do not target how the stretch frequency and velocity affect the linearity of the sensor over time.

The stretch sensors inherit an inconsistency of measurements due to displacing particles inside the sensor when stretched. It is unlikely to have the same resistance recording in each attempt from the same place; this is due to length deformations of bands. Using resistive bands for long-term applications like sleep monitoring could be a challenging task if exact behaviour is unknown. In this paper, we have used a stretchable conductive fabric (SCF) made with silver-coated fibres. The fabric can be used as an antibacterial wound or burn dressing, as well as electrode contact and shielded garment [30]; therefore, it is suitable for long-term vital monitoring. The fabric is stretchable in both horizontal and vertical directions and changes the internal resistance in both directions.

Since there is no perfectly linear band, a careful quantification method needs to be implemented to fully understand their internal characterisations and define error and biases that are correlated with their use. In this case, electrical and mechanical parameters are well observed and studied during stretching which provides all the necessary data about pre-calibration and compensates for the possible nonlinearities. This is beneficial to enhance band accuracy and performance, and ultimately become the benchmark for future results and experiments.

## 2. Materials and Methods

The parameters of the characterisation experiments were selected based on targeted physiological functions and the primary goal is to characterize the sensor based on parameters listed on Table 1.

### 2.1. Stretchable Fabric Bands

The electro-resistive sensor in this experiment was made of high ionic silver-plated nylon elastic knit in a double direction from Holland Shielding (Dordrecht, Netherlands). The manufacturer claims that the fabric manufactured with medical grade silver and suitable to be used directly on the skin. The fabric is rated for temperature from −30 ${}^{\circ }C$ to 90 ${}^{\circ }C$; therefore, it is safe to use in skin touch garments. All of the experiments were conducted in a controlled room environment with the temperature at 25 ${}^{\circ }C$ to avoid drifting caused by temperature fluctuations. Table 2 summarizes the fabric properties in raw format.

The choice of commercially available conductive fabrics is encouraged by the consistency of specifications between different samples compared to in-house research conductive fabric. The fabric can be purchased as rolls and easily cut into desired thickness and shape based on the application.

### 2.2. Weaving Structure and Change of Conductivity

**Figure 1:**
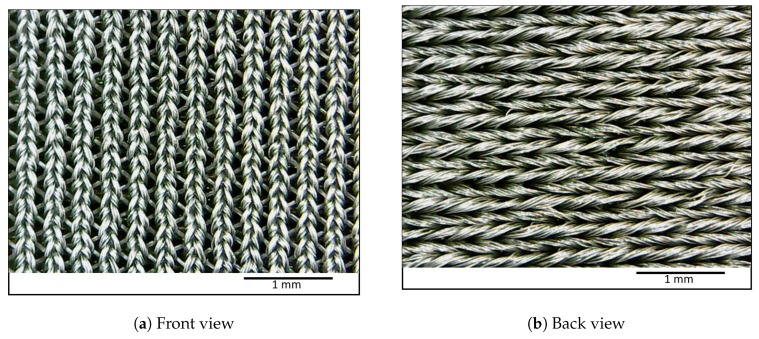
Unstretched fabric as viewed from the front and back sides.

The fabric has a double knot weave structure to allow both horizontal and vertical stretching. Figure 1a shows the front view of the unstretched fabric, and Figure 1b shows the unstretched backside of the fabric. The weaving pattern is arranged such that, during the horizontal stretch, the back threading get loosened while the front threading get tightened.

**Figure 2:**
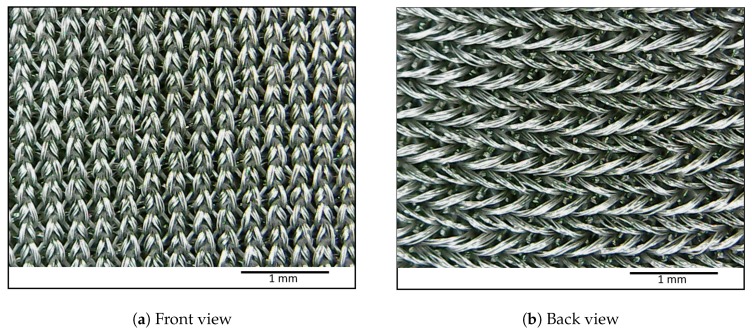
Tightening of front side threads and relaxation of back side threads of the fabric under the horizontal stretch.

When thin strips of bands are cut in a horizontal direction, the strips have predominant horizontal stretch motion. As shown in Figure 2, the micro-conductive gaps between single threads which contribute to the overall conductivity get loosened in the backside of the fabric during the stretch. The front side threads get tightened in a vertical direction which adds only a small contribution towards the increased conductivity in a horizontal direction. Therefore, an increased resistance is observed when the band is subjected to a horizontal stretch. However, when the backside thread achieves maximum relaxation, the relaxation effect could not contribute to resistance increase while the front side threads could tighten further. The tightening of the front side threads introduces increased conductivity in this scenario. By observing the weaving pattern and how it tightened when subjected to linear stretch, we could predict how resistance would change during different phases of stretch.

To understand the material limitations, we have tested them with different stretch length and recorded their internal resistance. The fabric sheet is cut into strips of 2 mm, 3 mm and 5 mm thin bands. The bands with identical dimensions show a small difference of initial resistance due to manufacturing or interfacing inconsistencies.

A length of 225 mm bands was used for all experiments. The bands were pre-stretched up to 10 mm to prevent slacking when fully contracted. As expected, bands with large width show lower initial resistance. For experiments that involve impedance linearity and impedance per unit length, the band resistance was measured by a Fluke 117 (Everett, WA, United States) digital multi-meter (DMM) [31] using the two-probe method (probe directly touching each end of the band) with ±0.9% + 0.2 Ω error. The length measurements were taken using a standard ruler with 1 mm resolution.

### 2.3. Simulating Respiration Output

**Figure 3:**
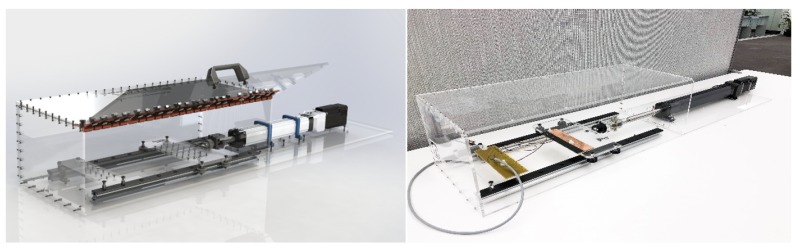
**Left:** A 3D visualization of the fully assembled rig made on SolidWorks 2018 software; **Right:** The real assembled rig made in “Maker-Space”, Western Sydney University, Penrith, Australia.

A human subject cannot be used in these kinds of quantitative experiments. A programmable linear actuator, Zaber A-BAR-E (Vancouver, Canada) [32] was used to stretch the band continuously and accurately. We designed and built an extraction and contraction supporting system and electrical interface for the linear actuator as shown in Figure 3. The actuator mimics the breathing operation of the human body by moving in a sinusoidal pattern in the same frequency range. The actuator has built-in stepper motor encoders and controllers that have a precision of 0.25 μm precision and a peak thrust of 540 N, therefore capable of precisely following the waveform for an extensive period.

This actuator is connected to a PC and controlled by an ASCII/Binary Protocol provided by the manufacturer. The custom-designed system that enclosed Zaber A-BAR consists of two V-slot linear rails of size 20 × 20 × 500 mm, with an additional thin and sharp edge linear rail that is used to avoid friction and potential vibration. The electrical interface board is connected to a ball joint to correct the actuator movement and keep the board aligned in a horizontal position. The electrical interface is made with a printed circuit board (PCB) with three channels connected to another PCB board with three channels via bands. The current travels from one board to another via bands and connects to the ground. The entire rig is designed inside a 6 mm thick acrylic box for safety purposes.

### 2.4. Electrical Interface

**Figure 4:**
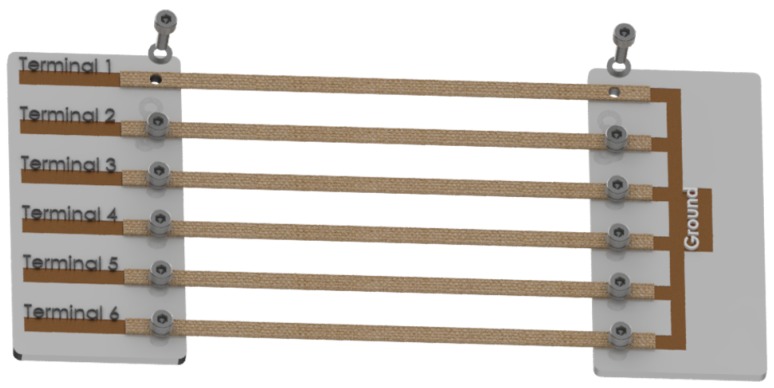
Disassembled view of band to stretch machine interface.

A 3D model of the PCB mounting plate is presented in Figure 4. A hole is cut into the band and mounted on to the PCB with nut and bolt with a washer. The connection is firmly tightened making a secure connection between the band and the movable plate.

**Figure 5:**
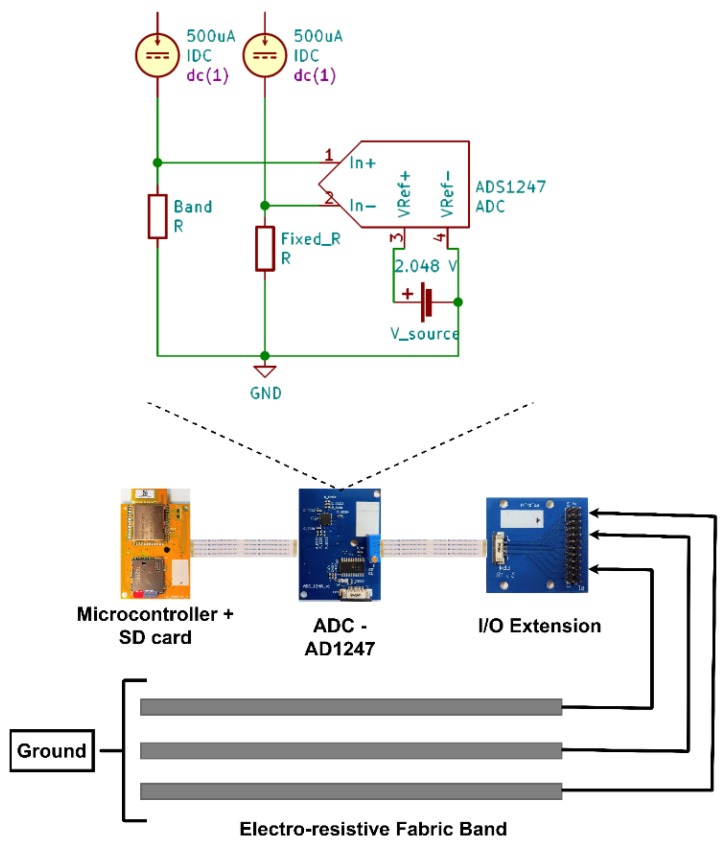
The analog to digital converter front-end used to measure the band resistance and the complete hardware configuration of data collection and data storage units.

The data acquisition system is designed and manufactured to cater the experiment requirements. As shown in Figure 5, the band and a fixed resistor are fed by a dual excitation matched current source. The fixed resistor is equivalent or slightly lower than the initial band resistance to introduce smaller bias and prevent saturation during the amplification stage. The ADS1247 [33] Analogue to Digital Converter (ADC) is directly connected to the band, and the fixed resistor via separate input channels to allow differential measurement.

A custom PCB that consists of ADS1247 ADC controlled by CC2640R2F micro-controller with an secure digital (SD) card slot was designed. The low power data acquisition system allowed for measuring and storing the experiment data with a battery powered isolated system. ADS1247 is a high-resolution 24-bit ADC capable of providing programmable current between 50 μA–1500 μA and programmable gain amplifier of factor 1–128. Each channel is sampled at 100 Hz with channel multiplexing between three bands, and captured data are stored in an 8 GB MicroSD card. The SD card data are transferred to a computer for data analysis after each experiment. The circuit is powered with two 1.5 volt AA batteries. The ADC gain is programmed as one, and the constant current is programmed to 250 μA.

### 2.5. The Evaluation Setup

The experiment has three main variables listed below:

1. Band width—2, 3, and 5 mm.
2. Iteration frequency—5, 10, 15, 20 and 30 cycles/min.
3. Stretch length—1, 2, 4, 8, 14 and 20 mm (peak-to-peak sine pattern).

We have chosen these iteration frequencies to be between 5 and 30 cycles/min because the study targets reproducing respiration. The normal respiration rate is between 10 and 20 cycles/min according to [34]. The stretch length is chosen to be between 1 and 20 mm to prevent the fabric band from reaching its permanent deformation point and avoid any possible damage for its physical and electrical properties. Every band was stretched less than 25% of its original length.

The output variables space is ninety different experiments on the band. Each fabric band (i.e., 225 mm × 2 mm, 225 mm × 3 mm, 225 mm × 5 mm) was tested thirty times on different frequency and stretch length. We started stretching the band from 1 mm to 20 mm and set the frequency to begin from 5 cycles/min to 30 cycles/min. We have observed the band output voltage that became stable after 1.5–2.5 min; therefore, each experiment was aimed to last for a minimum of five minutes. We also measured the initial and final length/resistance of the band before/after every experiment. The actuator output was compared with the internal rotary encoder to confirm a precise interpolation of the sinusoidal expansion/contraction. For every experiment, the data were saved and checked individually to ensure that the analogue signals were correctly converted and output voltage data were stored as a binary file on the SD card.

### 2.6. Data Processing and Analysis

Three identical (length and width) bands were tested together with channel multiplexing so that each experiment has three recordings. A total of 270 recordings were taken varying all the parameters. The experiment reading is in 24-bit to two’s complement format with the ADC reference at 2.048 V. All acquired data were read from an SD card using proprietary binary to American Standard Code for Information Interchange (ASCII) conversion software and transferred to the MATLAB software (MathWorks, Massachusetts, United States) environment for further analysis. The data were pre-processed to make all signals start from the same phase to efficiently compare their performance. The data analysis is based on the following parameters:

1. Baseline change,
2. Amplitude change,
3. Phase difference,
4. Correlation coefficient,
5. Total harmonic distortion,
6. Signal to noise ratio,
7. Hysteresis,
8. Repeatability.

The rationale of choosing each parameter is described in Section 4.1.

#### 2.6.1. Baseline Change

**Figure 6:**
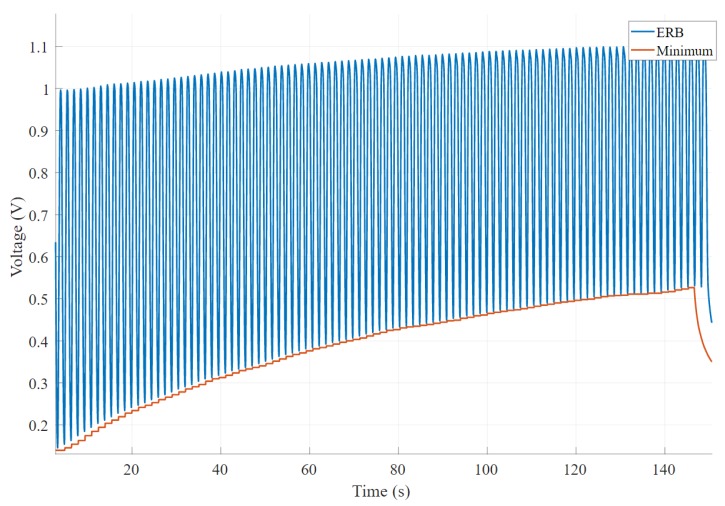
An example of undesired voltage output where baseline changes significantly with time.

**Figure 7:**
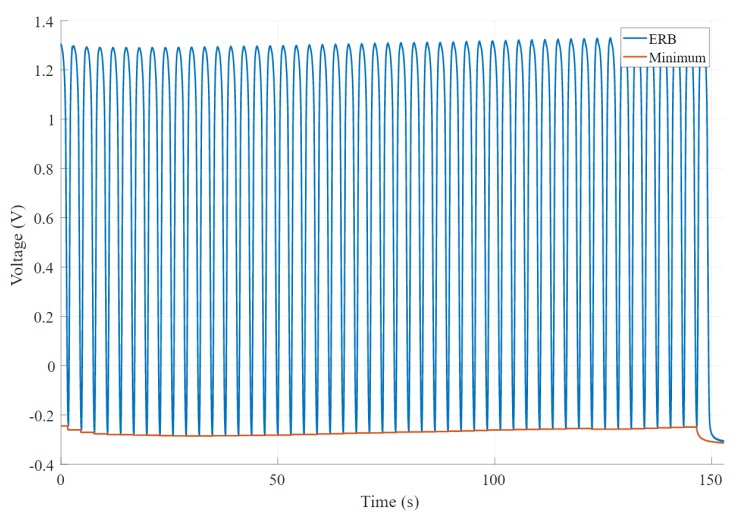
An example of desired voltage output where baseline remains relatively unchanged with time.

The baseline change is calculated by applying a sliding window function with size 1250 that returns the minimum value of each window. Then, we calculate the statistical information of the minimum value array such as minimum, maximum, standard deviation and mean. We take the difference between minimum and maximum of the applied sliding window to get the absolute baseline change. Figure 6 shows an example scenario chosen from 270 band recordings, where the output signal shows a significant baseline change, while Figure 7 illustrates a satisfactory baseline change.

#### 2.6.2. Amplitude Change

**Figure 8:**
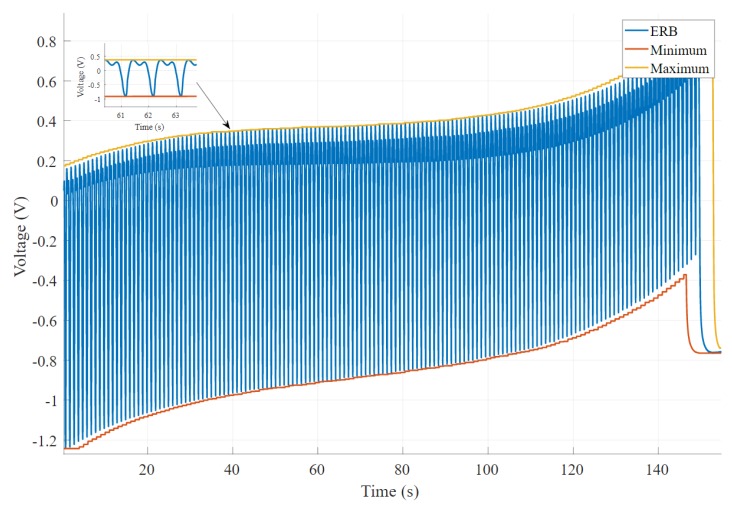
An example output of the band voltage where signal amplitude decreases significantly over time. The output also shows a high baseline change.

**Figure 9:**
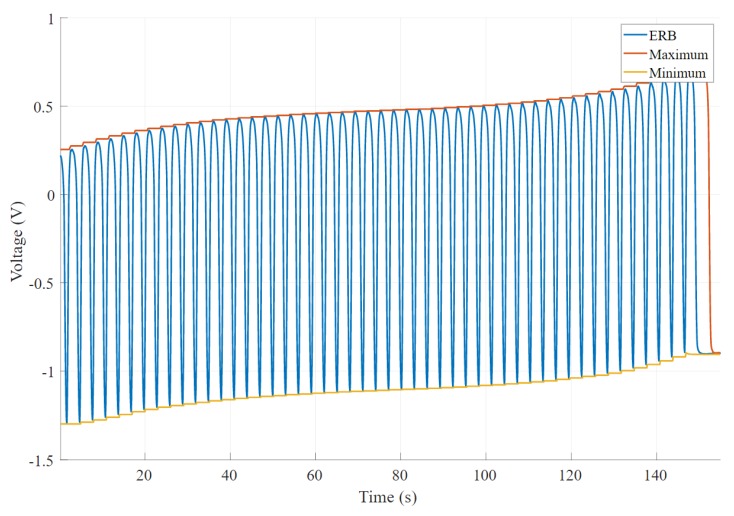
An example output shows a consistent amplitude. The graph shows considerable baseline changes; however, it does not affect the amplitude of the signal.

A moving window function has been implemented that returns both the minimum and maximum of the signal to get the peak-to-peak value as the difference between the two outputs [35]. Figure 8 shows an example of a signal chosen from 270 band recordings that changes the amplitude with time. It is clear that this signal became complex, and amplitude decreases with time. Figure 9 shows an experimental band that results in a minimum change of the signal’s amplitude. In this scenario, the baseline of the signal changes; however, amplitude remains the same.

#### 2.6.3. Phase Difference

Phase difference is calculated by normalizing the output data and taking the cross-correlation between normalized data, and frequency matched sinusoidal signal with zero phase. The highest cross-correlation value used to calculated the lag in number of samples. Since sample rate and frequency is known, the phase difference angle can be calculated in degrees.

#### 2.6.4. Correlation Coefficient

**Figure 10:**
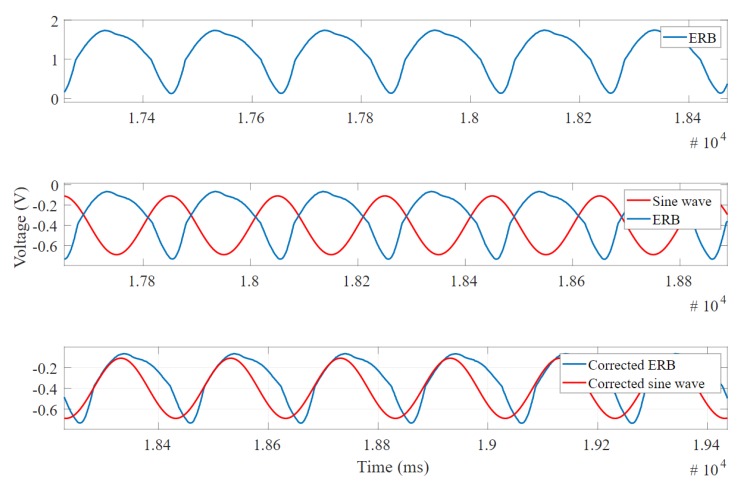
The pre-processing steps performed on the signal to apply cross correlation.

The phase difference only gives information about the fundamental frequency. The correlation coefficient is considered as a measure to represent the similarity between the sine wave and the band wave depending on the amount of error, phase difference and noise. Figure 10 illustrates the pre-processing steps applied before calculating the correlation coefficient.

Figure 10 shows the cross-correlation application and it makes the phase difference between reference and signal to be minimum. Then, the phase-corrected signal is used to calculate the phase difference and correlation coefficient. The phase difference was calculated using Discrete Fourier Transform (DFT) and taking the angle between both of the fundamental frequencies.

#### 2.6.5. Total Harmonic Distortion (THD)

Total Harmonic Distortion is an important figure of merit used to quantify the level of existing harmonics in any type of waveform [36]. If the bands are linear and the actuator provides a accurate sine wave, the band output voltage should appear as a similar sine wave pattern. In contrast, having a sinusoidal wave as an input, most of the experiment output from the bands are multi-frequency wave or a complex wave, which contains the fundamental frequency component of the input signal and an associated range of harmonics. The THD is measured by measuring the total energy which appears at the output of the system at harmonics of the input frequency [37]. The total harmonic distortion is approximately the square of the total signal’s power, which is the sum of the first five harmonics divided by the fundamental frequency using a modified periodogram of the same length as the input signal. Equation (1) shows the equation to calculate the total harmonic distortion [36] by dividing the energy of first five harmonic by the fundamental frequency:

(1) $$THD=\frac{\sqrt{H_{1}^{2}+H_{2}^{2}+H_{3}^{2}+H_{4}^{2}+H_{5}^{2}}}{Fundamental_{Frequency}},$$

where THD is the total harmonic distortion in decibels, and $H_{1-5}^{2}$ is the power of first five harmonic signals.

**Figure 11:**
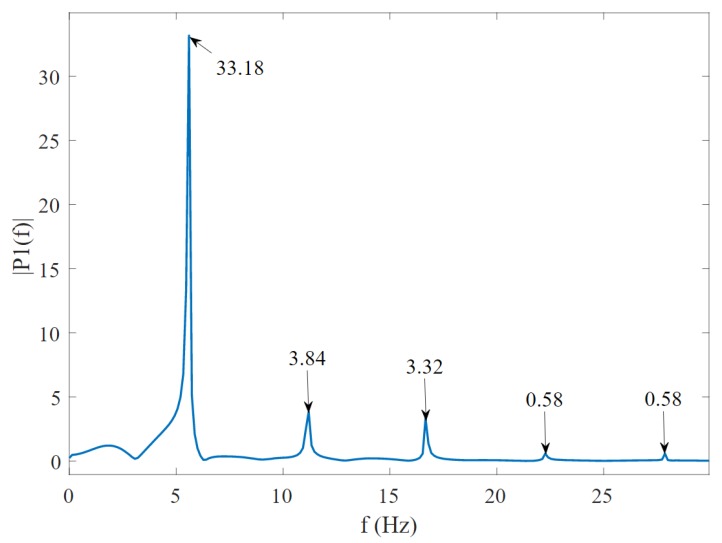
The Fast Fourier Transform on the signal to compute total harmonic distortion (THD) showing fundamental frequency and harmonics.

In Figure 11, we have visualized the value of the fundamental frequency and the contribution from subsequent harmonics. These values are used in Equation (1) and then multiplied by 100 to calculate the THD percentage.

#### 2.6.6. Signal-to-Noise Ratio (SNR)

The signal-to-noise ratio measures the power ratio between the signal and the noise. The signal is a sinusoidal waveform. The noise is assumed as the band-stop filtered voltage readings from the bands where the fundamental frequency is removed. First, we compute the Fast Fourier Transform (FFT) of the band signal (Data) and select their absolute values from FFT output. Equation (2) shows the discrete fourier transform (DFT) equation [38], which is defined for a vector *x* with *n* uniformly sampled:

(2) $$y_{k+1}=\sum_{j=0}^{n-1}\omega^{jk}x_{j+1},$$

where $\omega =e^{-2\pi i/n}$ is one of *n* complex roots of unity where *i* is the imaginary unit. For *x* and *y*, the indices *j* and *k* range from 0 to n−1.

Secondly, we remove the zero frequency signal from FFT output and take the frequency that produces maximum value corresponding to the fundamental frequency (Fmax). Thirdly, the fundamental frequency amplitude and the full FFT output signal are raised to the power of two and subtracted to get the Noise component. Finally, we calculate SNR as the base 10 logarithms of the ratio between fundamental frequency power and noise power. Equations (3)–(5) illustrate the calculation steps to get the SNR value in the decibel log scale:

(3) $$SignalPower=2*Fmax^{2},$$

(4) $$Noise=\sum (FFT{\left(Data\right)}^{2})-SignalPower,$$

(5) $$SNR=10Log_{10}\frac{SignalPower}{Noise}\left(\mathrm{dB}\right).$$

In SNR calculation, the THD component is already present. However, the significance of the THD component can vary across experiments. For example, a high THD value and low SNR signal are not necessarily undesirable. The THD effect could be easily removed to achieve high SNR. However, if the SNR is low when THD value is also low, it shows that the noise is appearing as non-harmonic noise sources such as power line frequency and noise introduced during data acquisition.

#### 2.6.7. Hysteresis

Hysteresis is the lag in measurement due to the direction of variation of the entity being measured. It adds a bias to the measurement and helps in finding a deformation between contraction and extraction of the fabric band. The hysteresis curve represents the difference between the values of one variable depending on the direction of change of another variable. Therefore, we have analysed the hysteresis in our system against four distinct parameters:

1. Low iteration frequency (5 cycles/min) and low stretch length (0.5 mm),
2. Low iteration frequency (5 cycles/min) and high stretch length (14 mm),
3. High iteration frequency (30 cycles/min) and low stretch length (0.5 mm),
4. High iteration frequency (30 cycles/min) and high stretch length (14 mm).

#### 2.6.8. Accuracy

Accuracy includes the effects of errors due to component tolerance. In every experiment, we have identified the minimum parameter to be considered to enable our system to produce accurate results, and they represent the limit of our experiments. In Table 3, we have presented the minimum resistance that can be measured by the ADC circuit with a gain of 1. The rig is also capable of stretching the fabric band as low as 0.25 μm for the linear actuator and 1.73 μm for the encoder.

#### 2.6.9. Repeatability

The target application of characterizing fabric sensors is to use it to monitor the body volume change. Ultimately, ERBs are supposed to be worn continuously, and they are expected to produce results continuously. In this scenario, we have attempted to measure the repeatability of each sine wave motion against all of the following sine wave in the data recording.

Let *N* sine motions of frequency *f* and amplitude *A* recorded in a continuous recording. The maximum normalized cross-correlation function is used as the similarity function where output is 0–1, where 1 is the perfect match. For each sine motion *S*, the maximum cross-correlation $M_{n,m}$ where *M* is the cross-correlation of $S_{n}$ and $S_{m}$ calculated. For each sine motion, the correlation function was applied to result in a vector *V*,

$$V_{1}=\left[M_{1,1},M_{1,2},\ldots ,M_{1,N}\right]$$

$$V_{2}=\left[M_{2,2},M_{2,3},\ldots ,M_{2,N}\right]$$

$$V_{3}=\left[M_{3,3},M_{3,4},\ldots ,M_{3,N}\right]$$

⋮

$$V_{N-1}=\left[M_{N-1,N-1},M_{N-1,N}\right]$$

$$V_{N}=\left[M_{N,N}\right].$$

The operation is similar to auto-correlation, where the time lag is equal to the motion period. Each $V_{n}$ vector represents the repeatability of each wave cycle. Four different sinusoidal motion experiments were used to calculate repeatability in four distinct scenarios:

1. Low iteration frequency (5 cycles/min) and low stretch length (0.5 mm),
2. Low iteration frequency (5 cycles/min) and high stretch length (14 mm),
3. High iteration frequency (30 cycles/min) and low stretch length (0.5 mm),
4. High iteration frequency (30 cycles/min) and high stretch length (14 mm).

#### 2.6.10. Washability

The washability is an important measure for quantifying ERBs as it shows how well the ERB can handle being washed without affecting its internal structure and electrical properties. We used a domestic laundry washing machine and drying procedures to conduct wash tests to imitate real world application. One metre samples were used for wash tests with 3 mm width. After each washing attempt, we measured the resistance by placing the digital multi-meter probe on each end of the ERB and pre-stretch by 5%. We also measured the ERB length and captured the microscopic images from the top and side view of the band to show the internal structure after washing attempts.

## 3. Results

Three bands were tested for fracture length by stretching to the maximum until the bands are broken. The recorded maximum length is shown in Table 4. The results show that the band can be stretched to approximately four times their original length before being permanently damaged.

**Figure 12:**
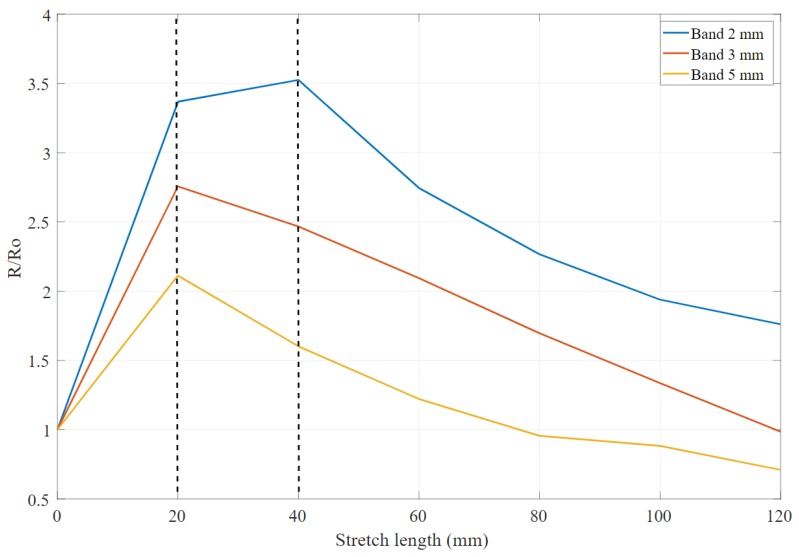
The value of the resistance ratio for each band after stretching up to 120 mm.

Figure 12 shows the resistance ratio of the bands after stretching it from 20 mm up to 120 mm. The 5 mm band resistance keeps increasing even until 40 mm stretch length equivalent to 33% of original length, whereas the 2 mm and 3 mm resistances start to decrease after a stretch length of 20 mm, which is equivalent to 17% of the original band length.

**Figure 13:**
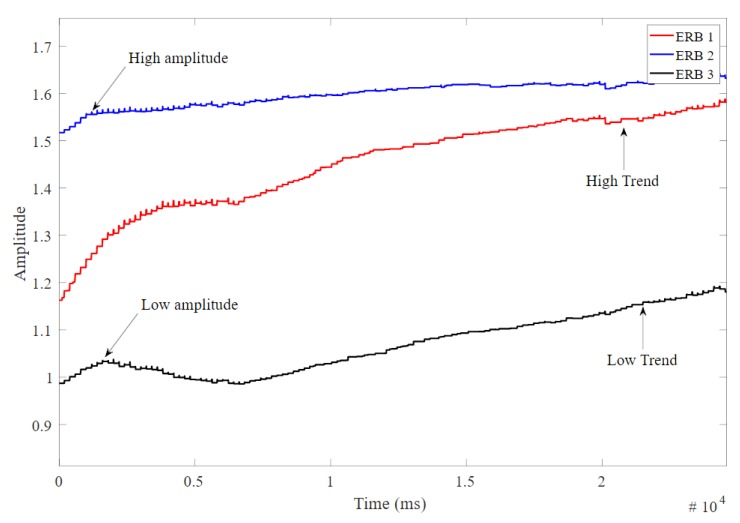
The amplitude change over time for three different band signals.

Figure 13 shows the amplitude change of three ERBs selected from the experiment to show non-correlated behaviour between amplitude change and initial amplitude. The amplitude and amplitude change are calculated as described in Section 2.6.2. A high amplitude (relates to higher stretch or thin band) could show low amplitude change over time, and low amplitude signal could show higher amplitude change over time.

**Figure 14:**
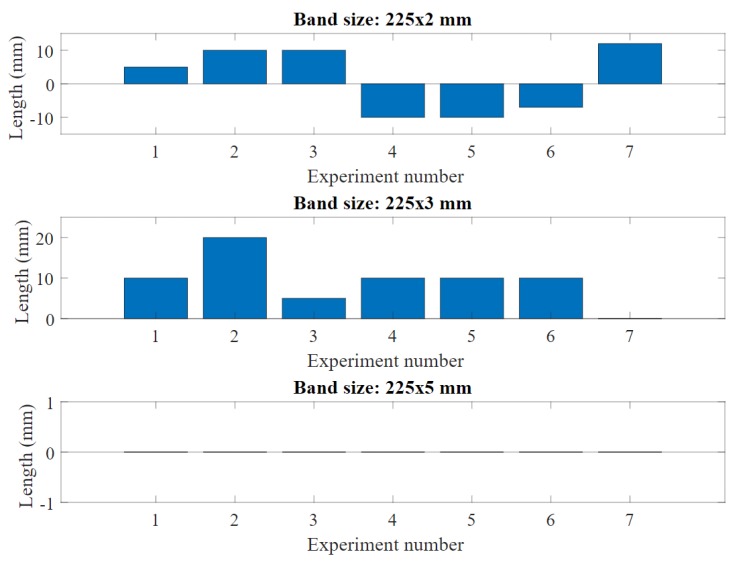
The length changes for all bands used in the experiment.

**Figure 15:**
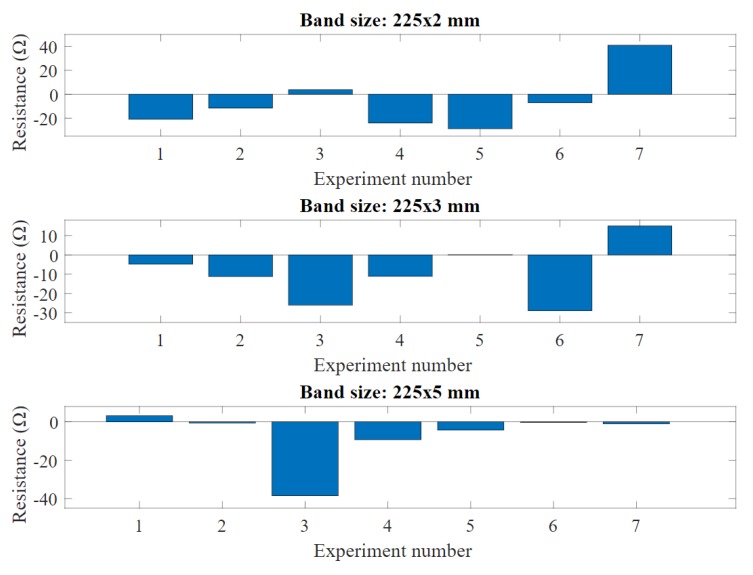
The resistance changes for all bands used in the experiment.

A data excerpt from fabric bands revealing changes in resistance and length after all the experiments is shown in Figure 14 and Figure 15. We found that the band resistance tends to change in some cases after multiple stretches, however without a significant difference between the initial and final measures overall (an average of only 23.6 Ω difference). Similar to the band resistance, the mean length change between initial and final is 10.71 mm. However, experiments showed that the length stays unchanged when the band width is at 5 mm, which gives an indication that a wider band can maintain its physical properties better compared to narrow bands over time.

The band characterisation on simulated breathing was purely based on how to differentiate a desirable/undesirable output from the band. Among the measures chosen, a higher signal-to-noise ratio and coefficient correlation would result in a better band output. A low value for all other measures, low standard deviation for baseline change, low total harmonic distortion, low phase difference, etc. indicate a better band output.

Ten different measures were calculated for all experiments. The threshold for desired values was chosen as the median. This gave us the ability to classify the desired and undesired test scenario for each band. Each measurement was individually considered or combined to select for which scenarios (frequency, stretch length and band width) the bands perform better to understand the overall desirable outputs.

**Figure 16:**
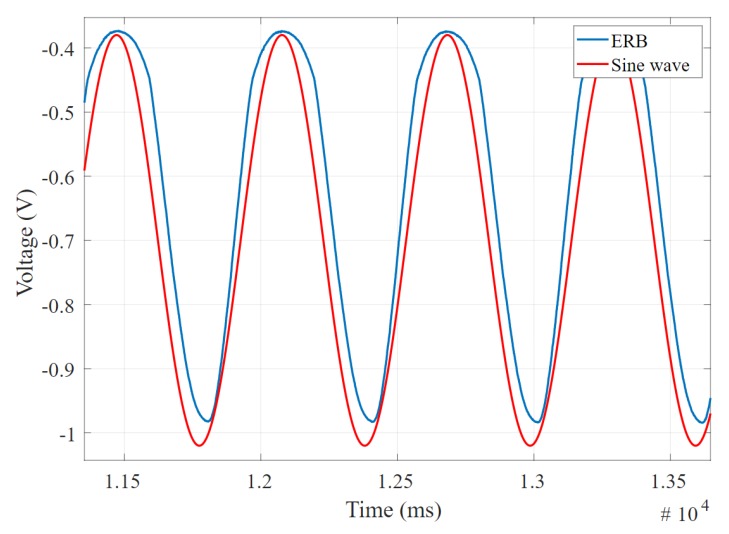
The electro-resistive band (ERB) output and expected sine wave showing low phase difference and high correlation coefficient.

**Figure 17:**
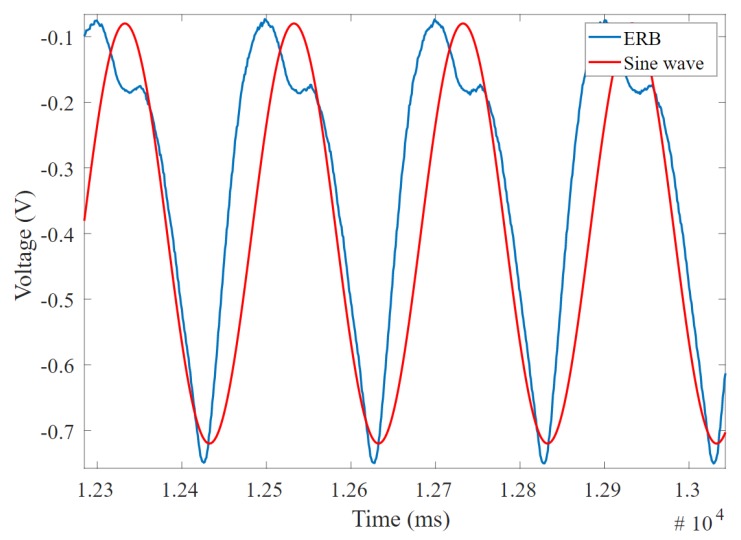
The electro-resistive band (ERB) output and expected sine wave showing high phase difference and low correlation coefficient.

Figure 16 shows the desired example of two signals that offer a low phase difference and high correlation coefficient, which indicate a good band for respiration application; on the other hand, Figure 17 shows an undesired example of two signals with high phase difference and low correlation coefficient, as well as complex waves and significant distortion on the signal peak. It is possible to have a low phase difference (which is desirable) but low correlation coefficient due to harmonic and high-frequency noise that makes some bands not useful for precise measurement applications.

Table 5 shows the median values for each parameter, and the normalized values mapped between –1 and 1. The median and normalized values are presented for amplitude change, standard deviation of the baseline value change (STD), signal-to-noise ratio (SNR), total harmonic distortion (THD) and correlation coefficient. Table A1, Table A2, Table A3, Table A4, Table A5, Table A6, Table A7, Table A8, Table A9, Table A10, Table A11, Table A12, Table A13, Table A14 and Table A15 show the parameters compared with cut-off median value. Each *H* cell presents a value that lies higher than the median, and *L* represents values lower than the median. The summary of results shows that, for low-frequency iteration, the band performance is better. This indicates that the bands are efficient and reasonably accurate for slow iteration. Having said that, slow iteration is closer to real-life experiments for human breathing (an average of 12–16 breaths each minute). We observed that the amplitude change is smaller for a wider band comparing it to the thin band; this means that a wider band is more capable of handling more different types of activities without drifting. The standard deviation of the baseline value increases with the frequency, making the standard deviation higher than the calculated median. Patterns show that bands that have a better output were subjected to slow frequency iteration between 5 and 15 cycles/min and a small amplitude stretch 1 to 4 mm. We also found that the phase difference tends to be smaller when the bands are subjected to high-frequency iteration.

**Figure 18:**
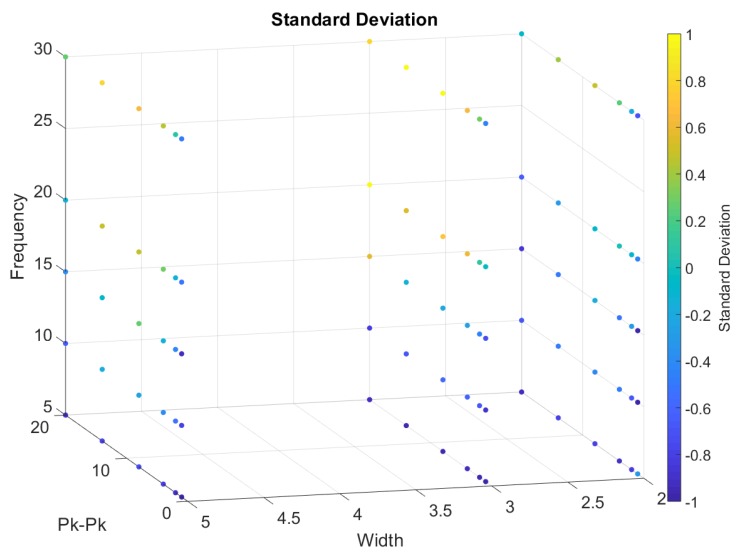
A 3D scatter plot for Standard Deviation.

**Figure 19:**
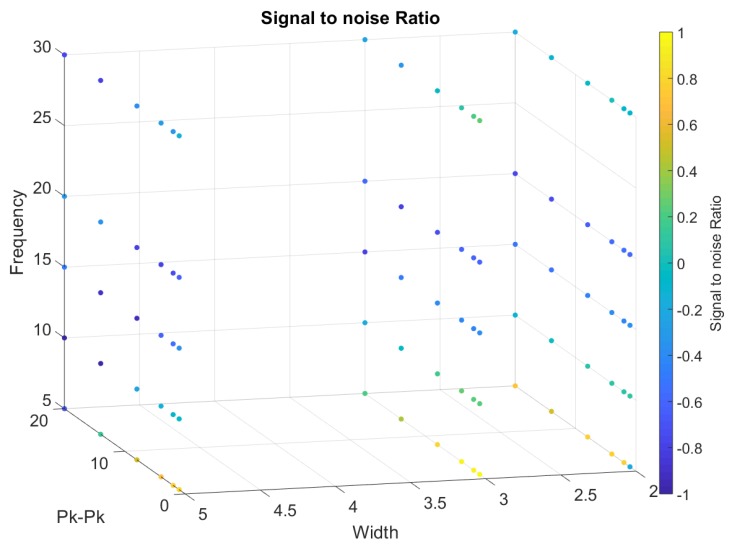
A 3D scatter plot for Signal to noise ratio.

**Figure 20:**
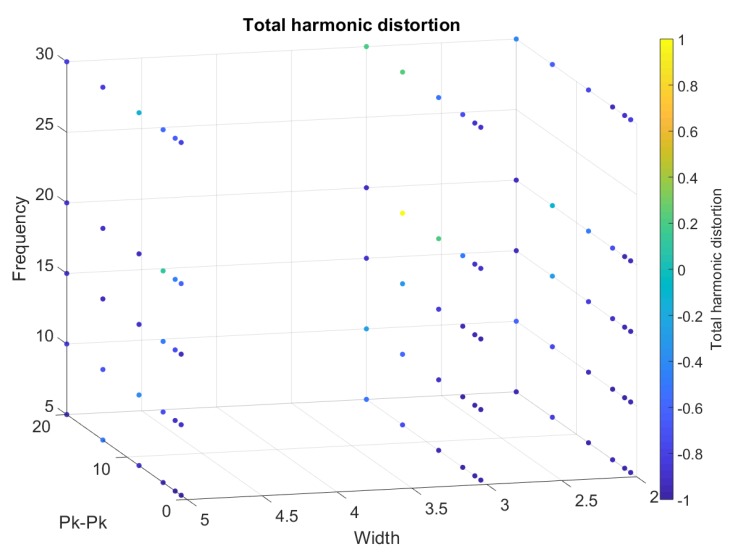
A 3D scatter plot for total harmonic distortion.

**Figure 21:**
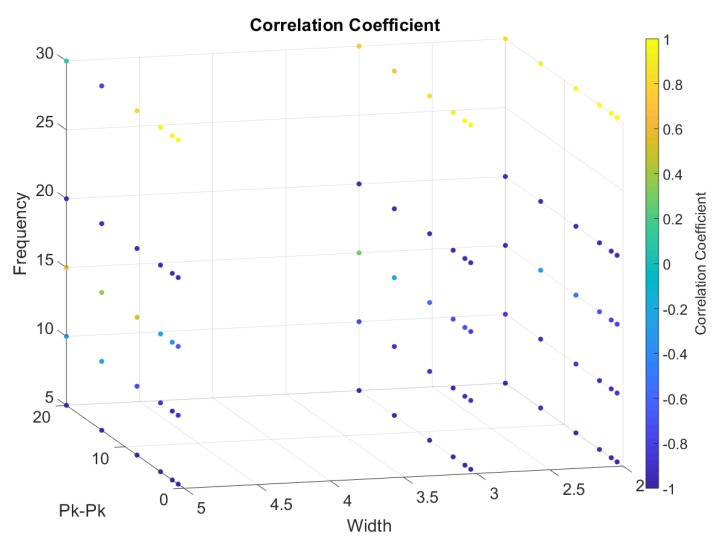
A 3D scatter plot for correlation coefficient.

**Figure 22:**
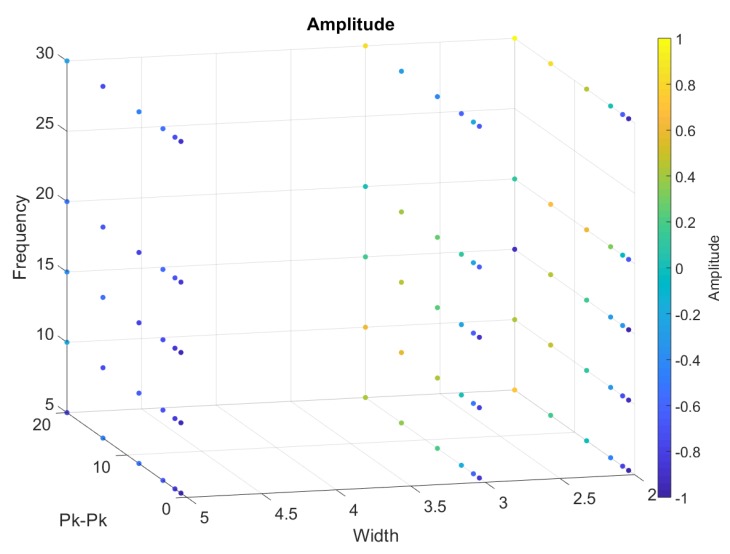
A 3D scatter plot for Amplitude.

From Figure 18, Figure 19, Figure 20, Figure 21 and Figure 22, we represented the final band results as a 3D scatter plot. We presented the absolute amplitude change of the signal, standard deviation of the baseline value change (STD), signal-to-noise ratio (SNR), total harmonic distortion (THD) and correlation coefficient. As stated earlier, a reliable band will have a small STD, THD, amplitude change and preferably a large SNR with a high correlation coefficient. Since there are three variables and one calculated value, the values are presented by the 4th dimension using colour. In addition, *x*, *y* and *z* axes of the scatter plot show the band width, iterating frequency and the stretch length. For every scatter plot, blue points show better results except for SNR and the correlation coefficient, which should be yellow. All the data are initially re-scaled between –1 and 1 for final evaluation and assessment. Results show that stretching the band between 1 mm and 5 mm is most suitable to eliminate nonlinearity and noise. Looking through the signal-to-noise ratio, the result shows that the band should be stretched with no more than 10 mm and with a low frequency between 5 to 10 cycles/min. Overall results show that a band with 2 mm width is more suitable to high stretch frequency but very limited by the stretch length.

Table A1, Table A2, Table A3, Table A4, Table A5, Table A6, Table A7, Table A8, Table A9, Table A10, Table A11, Table A12, Table A13, Table A14 and Table A15 show the variation across all the measurements for every stretch length. H is considered High for all values higher than the median in tables, and L is considered Low for all values lower than the median. The tables present the hard threshold cut-off value compared to the colour coded scatter plots for the same results.

**Figure 23:**
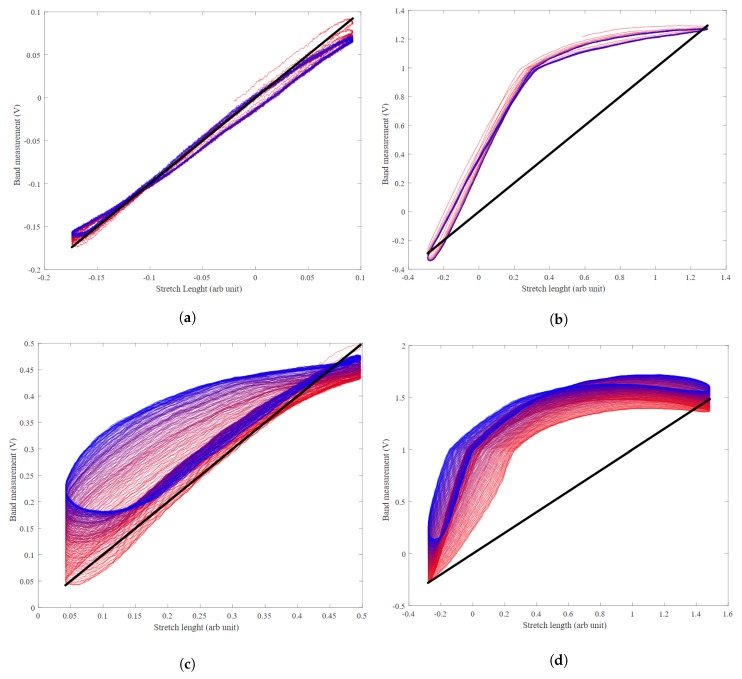
Hysteresis for a band with (**a**) a low iteration frequency (5 cycles/min) and low stretch length (0.5 mm), (**b**) a low iteration frequency (5 cycles/min) and high stretch length (14 mm), (**c**) a high iteration frequency (30 cycles/min) and low stretch length (0.5 mm), and (**d**) a high iteration frequency (30 cycles/min) and high stretch length (14 mm).

The hysteresis for four different sets of recording covering four possible scenarios shown in Figure 23. The diagonal straight line shows a no-hysteresis perfect scenario. The graphs, drawn from red to blue colour-coded, show how hysteresis changes from the start to the end of the recording. The red curves are from the start of the recording, and blue curves are at the end of the recording. The hysteresis graphs show two pieces of information. The first is how the amplitude nonlinearity appeared as the left-diagonal, the red colour to blue colour shift of the graphs. The second is the hysteresis as the deviation from the right-diagonal ideal line. As expected, the low frequency, low stretch bands show the lowest hysteresis. High iteration frequency introduces amplitude nonlinearity while both frequency and stretch result in higher hysteresis.

**Figure 24:**
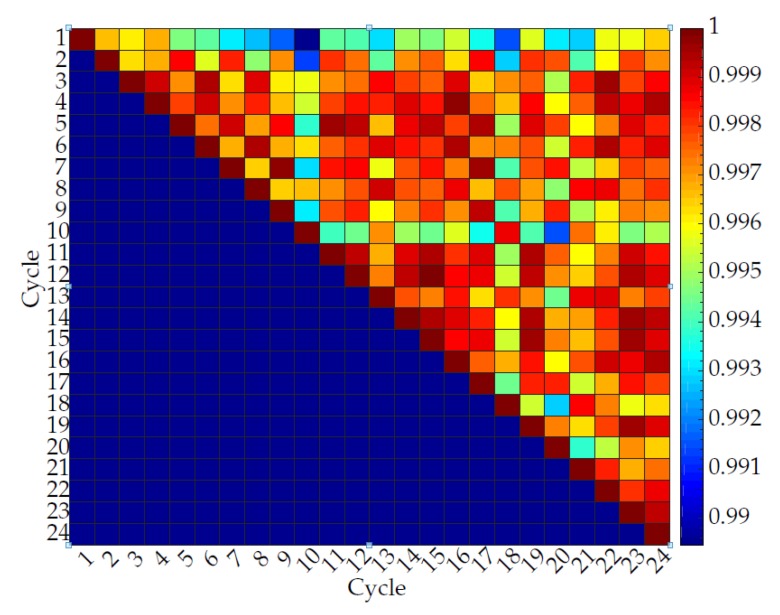
Normalized cross-correlation between sinusoidal motions for a band with a low iteration frequency (5 cycles/min) and low stretch length (0.5 mm).

**Figure 25:**
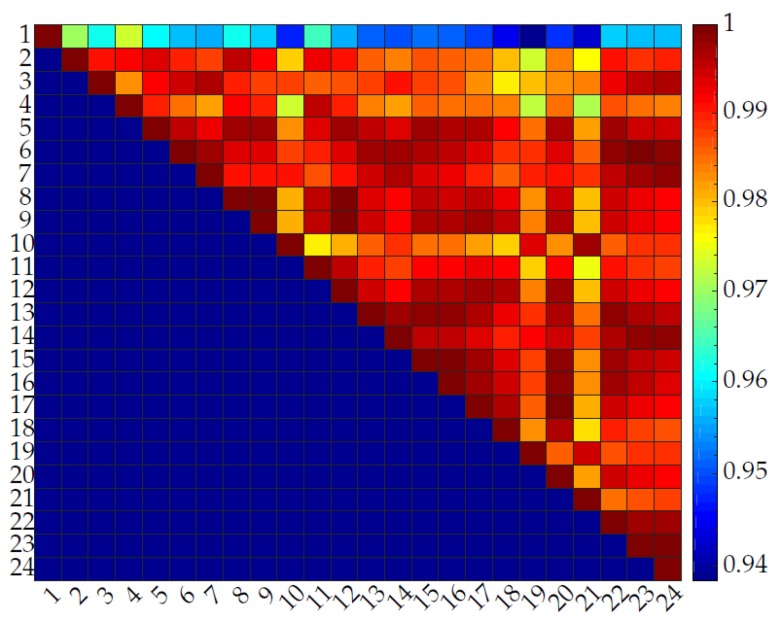
Normalized cross-correlation between sinusoidal motions for a band with a low iteration frequency (5 cycles/min) and high stretch length (14 mm).

**Figure 26:**
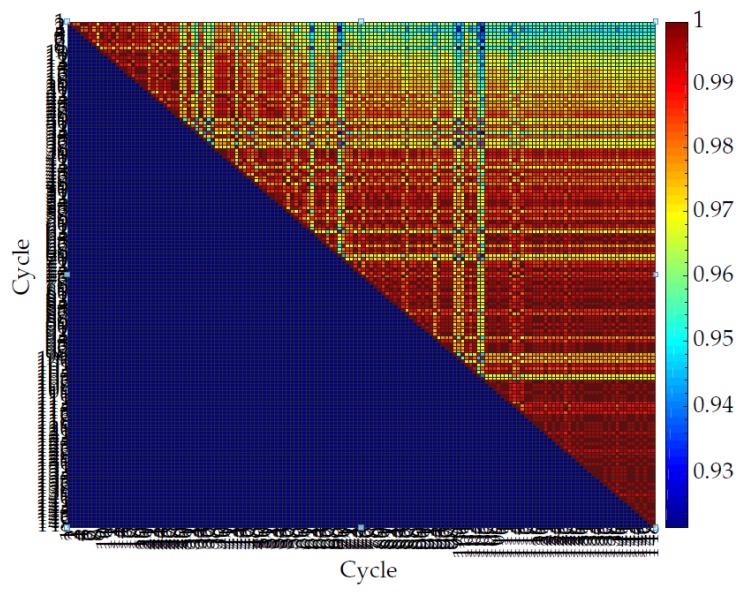
Normalized cross-correlation between sinusoidal motions for a band with a high iteration frequency (30 cycles/min) and low stretch length (0.5 mm).

**Figure 27:**
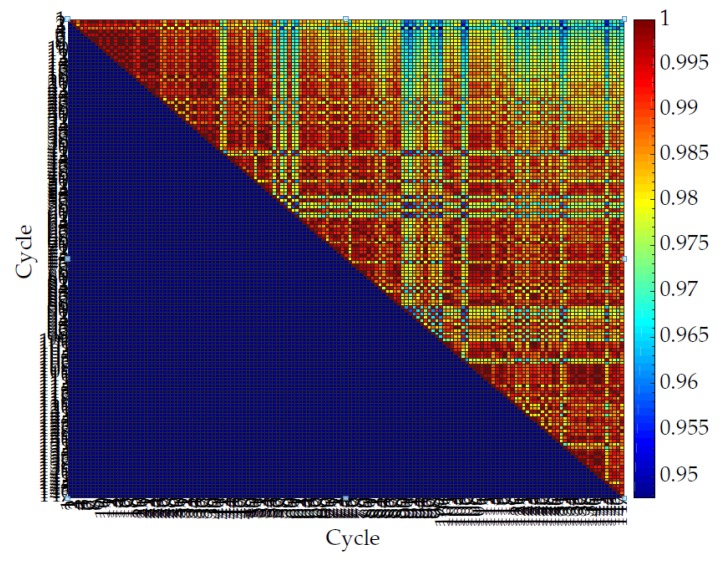
Normalized cross-correlation between sinusoidal motions for a band with a high iteration frequency (30 cycles/min) and high stretch length (14 mm).

Figure 24, Figure 25, Figure 26 and Figure 27 show the repeatability of the band output with four different edge cases. Each row of Figure 24, Figure 25, Figure 26 and Figure 27 represents the cross-correlation of the corresponding cycle against itself followed by subsequent cycles. The dark red represents a perfect match while dark blue represents a low correlation. The colour map is distributed to a log scale. Generally, the coefficient does not fall below 0.93 while showing high correlation (high repeatability). In addition, it is noticeable that the first few cycles are not repeatable as much as mid-end cycle values. Therefore, the fabric bands need to go through a few warm-up cycles to produce a stable output waveform and remain for a longer period of time.

**Figure 28:**
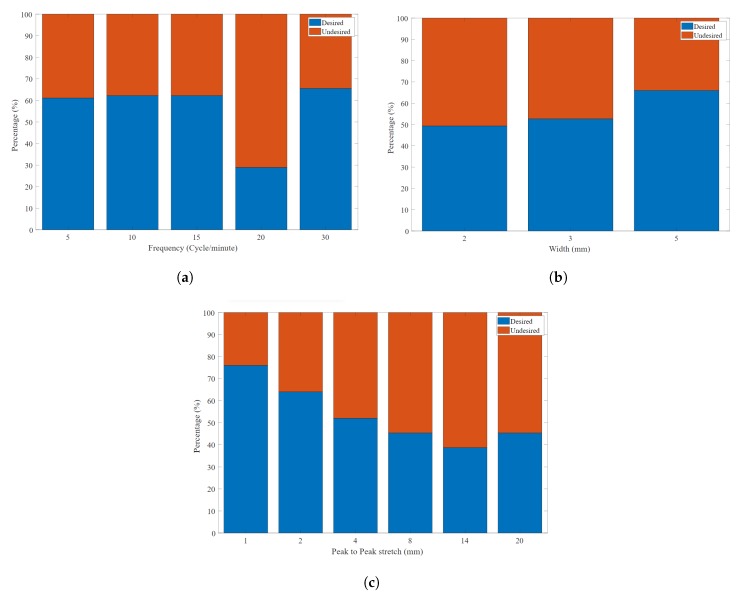
The desired and undesired experiment outcomes for (**a**) each frequency tested (**b**) each band width used (**c**) each stretch amplitude tested.

From Figure 28a–c, we show the desired value against the undesired values using the results obtained after threshold based binarization. Based on these readings, we have characterised the bands in terms of frequency (cycle per minute), band width (mm) and signal peak to peak (voltage). In terms of frequency, 30 cycles/min shows a high percentage of success with 65.5%. A band width of 5 mm is the highest desirable (66%) followed by the 3 mm band. Finally, in terms of the peak-to-peak stretch, 1 mm and 2 mm have the highest values with 76% and 64% respectively for the desired value.

**Figure 29:**
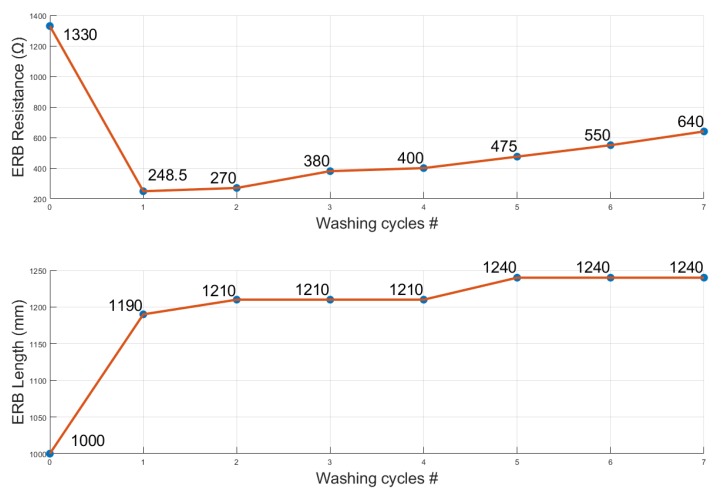
The ERB resistance and length after seven washing cycles.

**Figure A1:**
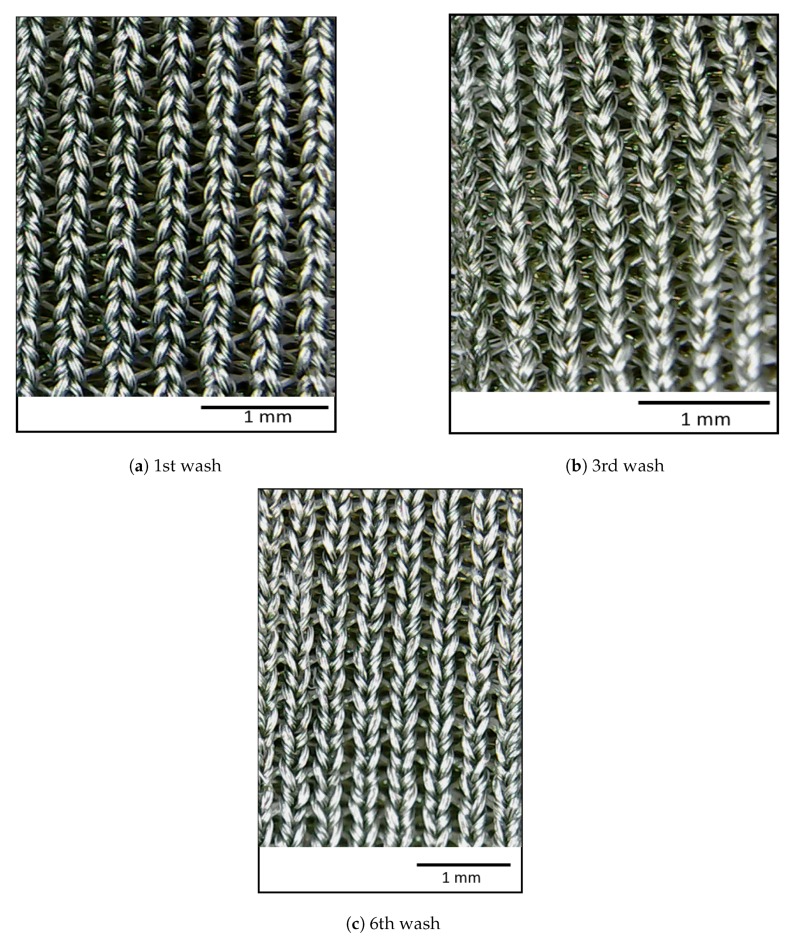
Front side microscopic figures after 1st, 3rd and 6th wash cycles.

**Figure A2:**
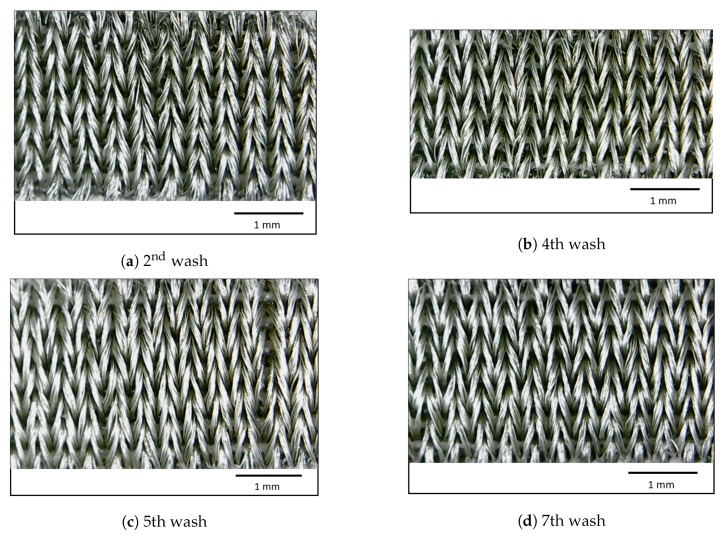
Back side microscopic figures after 2nd, 4th, 5th and 7th wash cycles.

In Figure 29, we show the results of the washability test. We performed seven washing experiments with the ERB focusing on resistance, length and appearance. Final results show that ERB resistance decreased from 1.3 kΩ to 248.5 Ω, and then it starts to slowly increase until it becomes 640 Ω in the 7th wash. The band length was initially 1 m long; however, after the first wash, the length became 1.21 m, and, after multiple washes, it slowly increases up to 1.24 m. The initial wash made the highest impact on the fabric and the test results conclude that the ERB internal structure with electrical characteristics become stable in subsequent washes. Figure A1 and Figure A2 show the microscopic images of the ERBs after each wash cycle for visual analysis. The internal structure of the ERB remained the same without any deformation or any major changes in the double weave knit pattern.

We can conclude that, in order to get the best bio-signal results, our SCF bands should be stretched within the parameter below:

1. Band width ≥3 mm.
2. Iteration amplitude ≤4 mm (≤2% elongation).

## 4. Discussion

This work highlighted some important factors that will impact the use of SCFs in biosensor devices to provide an accurate and reliable monitoring system. Our experiments aimed to find all calibration parameters needed to omit vibration, nonlinearity and signal corruption by choosing the correct band size, stretch length and acceptable frequency of stretch. We also found that the internal characteristics of the SCFs degrade if they are overstretched, as they will reach the permanent deformation phase. To assess the band performance, we designed and built a breathing simulator rig formed by a high torque linear actuator. Data captured from the sinusoidal waveform further processed to remove synchronization changes and obtained nine different parameters from each time-series signal. These parameters were later assessed together and separately to select the acceptable parameters that result in the best output. The reason to analyze parameters separately is because they are independent from each other and show the quality and performance of the band in a single dimension only. If some application desires one parameter over another, independent performance evaluation is useful. However, if the application needs to select a single band performing well across multiple parameters, the group analysis is useful.

### 4.1. The Rationale of Choosing the Characterisation Parameters

- **Baseline change:** The baseline change indicates the slow resistance drift of the fabric bands due to the change of weaving structure. The bands that have low baseline change are better because they need smaller bias adjustments in the data acquisition setup.
- **Amplitude change:** The amplitude change occurs when the band does not follow the same P-P output for the same amplitude stretch. It is preferable to have low amplitude change because fixed amplitude bands can be calibrated at the beginning for the length that would hold the calibration value for a longer period.
- **Phase difference:** The phase difference shows the output lag/lead compared to the input. A low phase difference is critical in applications where high time resolution is required. An example application is measuring pulse transit time using ERBs. A high phase difference would add uncontrolled variable into the measurement system which would eventually produce erroneous results.
- **Correlation coefficient:** The correlation coefficient shows the summing effect of phase difference, SNR, THD, repeatability and produces a parameter that describes the general quality of the band output. A high quality linear output would have a higher correlation coefficient.
- **Total harmonic distortion:** The total harmonic distortion analysed in our work focused only on first five harmonics produced by the band. We found that the band shows a strong first harmonic effect in some cases probably due to a double knit weaving structure competing for horizontal vertical resistance change. A simple harmonic filter would eliminate most of the harmonic effects from the band.
- **Signal-to-noise ratio:** The SNR describes the THD and any high frequency noise present in the system due to vibration, ADC and induced noise by power line. SNR is useful when bands are used in highly sensitive applications such as pulse extraction from the body.
- **Hysteresis:** The hysteresis is analysed in four edge cases in our work. The hysteresis presented in our paper shows the difference between the extraction and contraction curve with amplitude change over time. A useful example scenario of hysteresis characterisation is respiratory effort recordings. If two bands capture the inhale and exhale patterns from the chest and abdomen, a high hysteresis could severely distort the output. In such applications, a low hysteresis band is required.
- **Accuracy:** In our experiments, we used low power but high accuracy ADC, current sources to eliminate inaccurate results due to measurement errors. The extraction rig was chosen to be a high precision, high torque device.
- **Repeatability:** Repeatability shows the ability of the bands providing the same waveform continuously. Similar to hysteresis, the repeatability is presented in four edge cases here. We found out that the initial stretch cycle is not repeatable due to the effect of amplitude and baseline change; however, after 1–2 min, the cycles become highly repeatable.
- **Washability:** Washability tests show how the electrical/mechanical characteristics change with each wash cycles. One important finding of our study is that the impact of the first wash is very significant; therefore, if the fabric bands are subjected to wash, we would recommend washing the bands for at least one cycle before stitching/sticking into substrate garments. The other option is use a waterproof substrate to completely cover the bands to protect from washing effects.

SCFs behave differently during expansion and contraction, and it is apparent that the response and system linearity also change. When the fabric is stretched, the weaving structure gets deformed results in changes of both width and length simultaneously. Since the fabric resistance behaves differently for the two axes of movements, two different sine waves are convoluted together to make a complex sine signal as the output. Nonlinear resistance changes in the SCF are also due to non-uniform concentration of the conductive compound such as silver coating. We have assessed the ERB accuracy by taking into account all of the effect of errors from the components tolerance; the main components of our system were the high torque linear actuator from Zaber and the analogue to digital converter ADS1247; we have also tested our ERB on a small stretch length that was as small as the human hair width (approximately 25 um) providing clear output sine wave. We were also interested in studying the repeatability of the ERB by representing the cross-correlation of the corresponding cycle and measuring each sine wave pattern against all sine waves in the data recording. A band with low iteration frequency and high stretch length as well as high iteration frequency and high stretch length has amplitude nonlinearity and high hysteresis. To determine the durability of the band, we have also washed the ERB using the in-house washing machine multiple times and took microscopic images each time.

We have found that SCF can be used in smart clothing applications to monitor respiratory activities. We also found that, for respiratory applications, the band width should be larger than 3 mm and the band length should be chosen such as maximum respiratory volume change, which does not result in more than 2% elongation. The iteration frequency plays a minor role compared with band width and stretch amplitude considering continuous stretch motion applications. It is still possible to acquire calibrated respiratory volume without strictly adhering to these suggestions; however, there is a high possibility of acquiring a distorted or noisy signal for volume. For respiratory rate applications (which requires identifying the peaks only), the bands perform perfectly. Finally, after experimenting with the SCF for multiple washes, we found that the internal structure of the band doesn’t get affected by the water even after seven wash cycles. However, there is a change in the band resistance and length which became stable in subsequent wash attempts. Since the washing cycles performed without no substrate, we hypothesise that the bands would survive longer when covered with a waterproof substrate material.

## 5. Conclusions

We present experiments and calculations to evaluate performance of the stretchable conductive fabric using a mechanical stretchable rig and low power custom data acquisition system. We have assessed the performance and accuracy based on their consistency and data quality against noise, distortion and internal changes in resistance. These fabrics behave differently depending on the application that they are used for, and mainly due to their elongation difference in the width and length. However, a band with small resistance and subjected to small stretch length and iteration is the perfect band for measuring respiration rate and heart beat. A wider band shows lower resistance; therefore, it is more suitable for body volume measurement applications. If the application requires a linear response, the maximum stretch length should be less than 2%–4% of the initial length. The work conducted in this paper has the potential to expand the range of function that can be achieved in the bio-integrated system, with the potential utility in the accurate physiological monitoring system and noninvasive sensors such as monitoring respiration during sleeping as well as other types of activities that require a continuous respiration monitoring. Finally, our paper can be useful as a reliable reference for researchers in their future work in conductive fabrics, electro-resistive bands or similar materials.

## Figures and Tables

**Table 1:** Characterisation measures and intended use case.

Characterization Parameter	Usage
Impedance per unit length	Identify the bias point and maximize the dynamic range without saturating the analog to digital converter (ADC) input
Impedance linearity with length	Pre-calibrate the sensor for absolute measurements
Mechanical moving frequency response	Apply the sensor in wide variety of stretch conditions. Examples: breathing (low frequency high stretch), pulse (high frequency low stretch), activity (low to high frequency with low to high stretch)
Signal quality measurements Total harmonic distortion Signal to noise ratio Phase difference Correlation coefficient Hysteresis Repeatability	Assessing the signal quality to decide if the sensor is useful in given application.
Length change over time	Minimize the sensor slacking during long-term usage
Yield length	The maximum useful stretch limit
Fracture length	Secure the bands from breaking apart

**Table 2:** Material specifications.

Property	Value
Resistance	<0.5 Ω cm^−2^ while unstretched
Shield effect	35+ dB
Temperature range	−30 to 90 ${}^{\circ }C$
Thickness	0.4 mm
Stretch	100% × length; 65% × width direction
Permanent deformation	After ±30% stretch

**Table 3:** Minimum values for experiment parameters.

Parameter	Value
ADS1247	0.012 Ω (Gain = 1)
Stretch Length	0.25 μm
Encoder precision	1.73 μm

**Table 4:** The maximum stretch length electro-resistive bands (ERBs) can handle before entering the fracture length starting initially from 225 mm.

**Band** 2 mm	**Band** 2 mm	**Band** 2 mm
900 mm	960 mm	810 mm

**Table 5:** The median and normalized median for all measures.

	Amplitude Change	STD	THD	SNR	Correlation Coefficient
Median	21.99	1.15	−10.39	37.44	0.05
Normalized Median	−0.44	−0.38	0.25	0.54	−0.89

STD: Standard deviation of the baseline value change, THD: total harmonic distortion, (SNR) signal-to-noise ratio.

**Figure A3:**
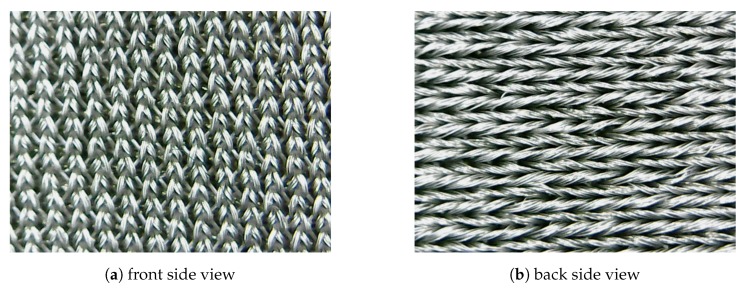
The microscopic view of the fabric after the experiment.

## Appendix A

### Appendix A.1.

The cut-off median value for five parameters, standard deviation of baseline change (STD), amplitude change (Amplitude), total harmonic distortion (THD), correlation coefficient and signal-to-noise ratio (SNR) are shown in Table A1, Table A2, Table A3, Table A4, Table A5, Table A6, Table A7, Table A8, Table A9, Table A10, Table A11, Table A12, Table A13, Table A14 and Table A15.

**Table A1:** Values higher and lower than the median for Frequency of 5 cycles/min for a 2 mm band.

Frequency 5 (Cycle/min)	1 mm	2 mm	4 mm	8 mm	14 mm	20 mm
STD	H	L	L	L	L	L
Amplitude	L	L	L	H	H	H
THD	L	L	L	L	H	L
Correlation Coefficient	L	L	L	L	L	L
SNR	H	H	H	H	H	H

**Table A2:** Values higher and lower than the median Frequency 10 cycles/min for a 2 mm band.

Frequency 10 (Cycle/min)	1 mm	2 mm	4 mm	8 mm	14 mm	20 mm
STD	L	L	L	L	L	L
Amplitude	L	H	H	H	H	H
THD	H	L	L	H	L	H
Correlation Coefficient	H	H	H	H	H	H
SNR	H	H	H	H	H	H

**Table A3:** Values higher and lower than the median Frequency 15 cycles/min for a 2 mm band.

Frequency 15 (Cycle/min)	1 mm	2 mm	4 mm	8 mm	14 mm	20 mm
STD	L	L	L	H	L	L
Amplitude	L	H	H	H	H	L
THD	L	L	L	H	H	L
Correlation Coefficient	H	H	H	H	H	L
SNR	L	L	L	L	L	L

**Table A4:** Values higher and lower than the median Frequency 20 cycles/min for a 2 mm band.

Frequency 20 (Cycle/min)	1 mm	2 mm	4 mm	8 mm	14 mm	20 mm
STD	L	H	H	H	L	L
Amplitude	L	H	H	H	H	H
THD	L	L	H	H	H	L
Correlation Coefficient	L	L	L	L	L	L
SNR	L	L	L	L	L	L

**Table A5:** Values higher and lower than the median Frequency 30 cycles/min for a 2 mm band.

Frequency 30 (Cycle/min)	1 mm	2 mm	4 mm	8 mm	14 mm	20 mm
STD	L	H	H	H	H	H
Amplitude	L	L	H	H	H	H
THD	H	L	L	H	H	H
Correlation Coefficient	H	H	H	H	H	H
SNR	H	H	H	H	H	H

**Table A6:** Values higher and lower than the median for Frequency of 5 cycles/min for a 3 mm band.

Frequency 5 (Cycle/min)	1 mm	2 mm	4 mm	8 mm	14 mm	20 mm
STD	L	L	L	L	L	L
Amplitude	L	L	H	H	H	H
THD	L	L	L	L	H	H
Correlation Coefficient	L	L	L	L	L	L
SNR	H	H	H	H	H	H

**Table A7:** Values higher and lower than the median Frequency 10 cycles/min for a 3 mm band.

Frequency 10 (Cycle/min)	1 mm	2 mm	4 mm	8 mm	14 mm	20 mm
STD	L	L	L	L	L	L
Amplitude	L	H	H	H	H	H
THD	H	L	L	L	H	H
Correlation Coefficient	L	L	L	L	L	L
SNR	H	H	H	H	H	H

**Table A8:** Values higher and lower than the median Frequency 15 cycles/min for a 3 mm band.

Frequency 15 (Cycle/min)	1 mm	2 mm	4 mm	8 mm	14 mm	20 mm
STD	L	L	L	L	L	H
Amplitude	H	H	L	L	L	L
THD	H	H	H	H	L	H
Correlation Coefficient	L	H	H	H	H	H
SNR	H	L	L	H	L	L

**Table A9:** Values higher and lower than the median Frequency 20 cycles/min for a 3 mm band.

Frequency 20 (Cycle/min)	1 mm	2 mm	4 mm	8 mm	14 mm	20 mm
STD	H	H	H	H	H	H
Amplitude	L	H	H	H	H	H
THD	L	L	H	H	H	H
Correlation Coefficient	L	L	L	L	L	L
SNR	L	L	L	L	L	L

**Table A10:** Values higher and lower than the median Frequency 30 cycles/min for a 3 mm band.

Frequency 30 (Cycle/min)	1 mm	2 mm	4 mm	8 mm	14 mm	20 mm
STD	H	L	L	L	L	L
Amplitude	H	L	L	L	L	L
THD	L	L	H	H	H	H
Correlation Coefficient	H	H	H	H	H	H
SNR	H	H	H	H	H	H

**Table A11:** Values higher and lower than the median for Frequency of 5 cycles/min for a 5 mm band.

Frequency 5 (Cycle/min)	1 mm	2 mm	4 mm	8 mm	14 mm	20 mm
STD	L	L	L	L	L	L
Amplitude	L	L	H	H	H	L
THD	L	L	L	L	H	L
Correlation Coefficient	H	H	H	H	H	L
SNR	H	H	H	H	H	L

**Table A12:** Values higher and lower than the median Frequency 10 cycles/min for a 5 mm band.

Frequency 10 (Cycle/min)	1 mm	2 mm	4 mm	8 mm	14 mm	20 mm
STD	L	L	L	L	L	L
Amplitude	L	L	L	H	L	H
THD	H	H	L	L	L	H
Correlation Coefficient	H	H	H	H	L	L
SNR	H	H	H	H	L	L

**Table A13:** Values higher and lower than the median Frequency 15 cycles/min for a 5 mm band.

Frequency 15 (Cycle/min)	1 mm	2 mm	4 mm	8 mm	14 mm	20 mm
STD	L	L	L	H	L	L
Amplitude	L	L	L	L	H	H
THD	L	H	H	L	L	L
Correlation Coefficient	H	H	L	L	L	L
SNR	H	H	L	L	L	L

**Table A14:** Values higher and lower than the median Frequency 20 cycles/min for a 5 mm band.

Frequency 20 (Cycle/min)	1 mm	2 mm	4 mm	8 mm	14 mm	20 mm
STD	L	L	L	H	H	L
Amplitude	L	L	L	L	L	H
THD	H	H	H	L	L	L
Correlation Coefficient	H	H	H	H	H	H
SNR	L	L	L	L	L	H

**Table A15:** Values higher and lower than the median Frequency 30 cycles/min for a 5 mm band.

Frequency 30 (Cycle/min)	1 mm	2 mm	4 mm	8 mm	14 mm	20 mm
STD	L	L	L	H	H	H
Amplitude	L	L	L	H	L	H
THD	H	L	L	L	L	L
Correlation Coefficient	L	L	L	L	L	L
SNR	H	H	H	H	L	L

### Appendix A.2.

Figure A1 and Figure A2 show the microscopic photos taken from both the front and back sides of the bands after each wash. Figure A3 shows the microscopic picture of a fabric band after being subjected to a series of experiments described in this paper.
